# Examining the Fish Microbiome: Vertebrate-Derived Bacteria as an Environmental Niche for the Discovery of Unique Marine Natural Products

**DOI:** 10.1371/journal.pone.0035398

**Published:** 2012-05-04

**Authors:** Laura M. Sanchez, Weng Ruh Wong, Romina M. Riener, Christopher J. Schulze, Roger G. Linington

**Affiliations:** Department of Chemistry and Biochemistry, University of California Santa Cruz, Santa Cruz, California, United States of America; Argonne National Laboratory, United States of America

## Abstract

Historically, marine invertebrates have been a prolific source of unique natural products, with a diverse array of biological activities. Recent studies of invertebrate-associated microbial communities are revealing microorganisms as the true producers of many of these compounds. Inspired by the human microbiome project, which has highlighted the human intestine as a unique microenvironment in terms of microbial diversity, we elected to examine the bacterial communities of fish intestines (which we have termed the fish microbiome) as a new source of microbial and biosynthetic diversity for natural products discovery. To test the hypothesis that the fish microbiome contains microorganisms with unique capacity for biosynthesizing natural products, we examined six species of fish through a combination of dissection and culture-dependent evaluation of intestinal microbial communities. Using isolation media designed to enrich for marine Actinobacteria, we have found three main clades that show taxonomic divergence from known strains, several of which are previously uncultured. Extracts from these strains exhibit a wide range of activities against both Gram-positive and Gram-negative human pathogens, as well as several fish pathogens. Exploration of one of these extracts has identified the novel bioactive lipid sebastenoic acid as an anti-microbial agent, with activity against *Staphylococcus aureus*, *Bacillus subtilis*, *Enterococcus faecium*, and *Vibrio mimicus*.

## Introduction

Traditionally, marine invertebrates have been a mainstay for the discovery of novel natural products scaffolds. In 2009 alone, 1011 new compounds were described from the marine environment, approximately half of which are of invertebrate origin. [1] More recently, attention has focused on the symbiotic and mutualistic microbial communities living within these sessile marine organisms. Careful examination of these communities using a variety of microbiological and molecular techniques is revealing that microorganisms are the true producers of many of the compounds first isolated from host invertebrates. [2], [3] Terrestrial microorganisms, especially those from the order Actinomycetales, formed the backbone for the golden age of antibiotic discovery, and are the source of the vast majority of FDA-approved antimicrobial agents. [4] In addition, whole genome sequencing has revealed that these organisms possess many more biosynthetic gene clusters than are predicted from the results of chemical analyses performed to date. [5] This suggests that there is an untapped opportunity for new compound discovery concealed within the genome sequences of Actinobacteria. For example, the genome sequence for *Streptomyces avermitilis* reveals gene clusters that code for the production of 37 secondary metabolites, yet only 13 natural products have been reported from this organism. [4]

In the ongoing search for new drug leads, it has been postulated that, in addition to activating silent biosynthetic gene clusters, emphasis on under-explored bacterial niche environments could also aide in the discovery of new chemical space. [6] Emphasis on these unexplored microenvironments has garnered significant attention among the natural product community over the last few years. Examples of these unique environments include hydrothermal vents and invertebrate hosts, such as nematodes. [7], [8], [9], [10] Along these same lines, researchers have examined Actinobacteria from dissected mud daber wasps, and uncovered 15 diverse strains of *Streptomyces* spp., one of which led to the isolation of a novel anti-fungal macrocyclic lactam. [11], [12] Separately, a different research program connected with the Philippine International Cooperative for Biodiversity Groups (ICBG) program has successfully isolated novel bioactive natural products from mollusks and cone snails by culturing symbiotic bacteria from the tissues of these gastropods. [13], [14], [15]

While the studies discussed above are focused on invertebrates and insects, recent results from the human microbiome project have inspired our laboratory to investigate marine vertebrates as a potential source of novel marine Actinobacteria. [16] Examination of the human intestinal microbial flora indicates that there are surprisingly few Actinobacterial strains present with most microbiome communities. In total, in a study of the intestinal bacterial communities from three different subjects, only 22 Actinobacteria sequences were uncovered, representing 0.2% of the total bacterial nonchimeric 16S rDNA. [17] Of the Actinobacteria sequenced, only seven phylotypes were represented, four of which had not previously been associated with the human intestinal microflora, suggesting that intestinal bacterial diversity is higher than previously described. [18] In addition, a number of recent studies examining the gut microbiota of wild and laboratory fish have shown that there is evidence for a core gut microbiota within single species, [19] and that bacterial distributions vary widely between different species. [20], [21], [22]

Comparison of the microbial diversity of environmental samples from three distinct sources (human gut, [18] ocean, [23] and soil [24]) indicates that the distributions of phyla found using 16S rDNA sequencing differ significantly between each of these environmental niches. Although low, each of these separate sources contained measurable levels of Actinobacteria, many of which share low sequence identity at the 16S rDNA level. Given that discrete environmental niches contain unique distributions of Actinobacteria, and that vertebrates have been largely ignored to date with respect to natural products discovery, we therefore elected to examine the fish microbiome with a focus on the isolation of culturable Actinobacteria for natural products discovery.

Although Actinobacteria are prolific producers of secondary metabolites, their role in the gut is poorly understood. Commensal Actinobacteria of the genus *Bifidobacterium* have been shown to regulate interlukin-10 (IL-10) production in healthy hosts. [25] IL-10 is an anti-inflammatory cytokine that has been implicated in protection against inflammation-driven host pathology in germ free mice. From the natural products perspective, small molecules have been shown to influence intercellular signaling networks, especially between microbial species. [26] In a murine gut microbiota study, it has been demonstrated that antibiotics heavily modify intestinal microbial populations, and that after treatment with antibiotics, individuals are more susceptible to infection by pathogenic bacteria, highlighting the role that commensal bacterial populations play in controlling disease progression. [27]

We now present the first report of the examination of the fish microbiome as a source of microbial diversity for natural products research. This study has resulted in the isolation of taxonomically distinct Actinomycetales from fish intestines, as well as unique strains of Firmicutes and Proteobacteria. These bacteria display a wide range of biological activities against both Gram-positive and Gram-negative bacterial pathogens, and are able to inhibit the growth of a number of commercially important fish pathogens. Examination of the chemical extracts of liquid cultures of these organisms has revealed the presence of a new bioactive lipid, sebastenoic acid (**1**), which demonstrates that fish gut-associated bacteria can serve as a novel niche for discovering bioactive small molecules.

## Results and Discussion

**Figure 1:**
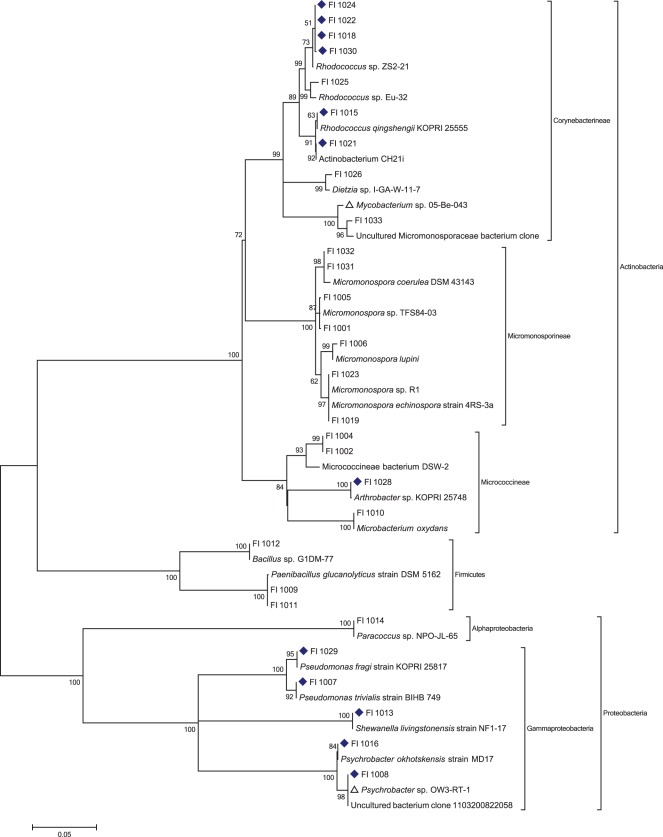
Molecular Phylogenetic Analysis by Maximum Likelihood for all Isolated, Culturable Strains of Bacteria Isolated from Fish Intestines. The evolutionary history was inferred by using the Maximum Likelihood method based on the Kimura 2-parameter model. [48] The bootstrap consensus tree inferred from 2000 replicates is taken to represent the evolutionary history of the taxa analyzed. [49] Branches corresponding to partitions reproduced in less than 50% bootstrap replicates are collapsed. The percentage of replicate trees in which the associated taxa clustered together in the bootstrap test (2000 replicates) are shown next to the branches. [49] Initial tree(s) for the heuristic search were obtained automatically as follows. When the number of common sites was <100 or less than one fourth of the total number of sites, the maximum parsimony method was used; otherwise BIONJ method with MCL distance matrix was used. A discrete Gamma distribution was used to model evolutionary rate differences among sites (5 categories (+*G*, parameter = 0.4869)). The rate variation model allowed for some sites to be evolutionarily invariable ([+*I*], 38.9340% sites). The tree is drawn to scale, with branch lengths measured in the number of substitutions per site. The analysis involved 53 nucleotide sequences. All positions containing gaps and missing data were eliminated. There were a total of 1244 positions in the final dataset. Evolutionary analyses were conducted in MEGA5. [30] Strains identified as psychrophilic bacteria in NCBI denoted with blue diamonds. Strains whose closest published NCBI relatives are uncultured clones denoted with open triangles.

Intact whole fish were purchased from commercial vendors based on local availability, or provided by collaborators (Table 1). Dissection under sterile conditions and plating of stomach and intestine contents onto solid agar media designed for the enrichment of Actinobacteria lead to the isolation of 29 bacterial strains (Table 1). 16S rDNA sequencing was performed using complimentary forward and reverse bacterial primers, with near full length 1400 bp sequences acquired for all isolates. Full sequences were edited with FinchTV (v. 1.3.1), assembled using CAP3 [28] followed by alignment with clustalX, [29] and phylogenetic trees built using Mega5. [30] (Figure 1).

**Table 1:** Fish Origins and Taxonomy, Microbial Isolates, and NCBI Closest Relatives.

Fish Source	Dissection Site	Isolate #	Accession #	Closest NCBI relative, Accession #	% Identity
Canadian Rock Cod,Santa Cruz	Stomach	1004	JQ691555	Micrococcineae bacterium DSW-2, FM995611.1	**97.8**
		1002	JQ691561	Micrococcineae bacterium DSW-2, FM995611.1	**97.8**
	Midintestine	–	–	–	–
	Posterior Intestine	1001	JQ691544	*Micromonospora* sp. TFS84-03, HM001288.1	99.9
		1006	JQ691560	*Micromonospora lupini*, AJ783995.1	99.2
		1005	JQ691559	*Micromonospora* sp. TFS84-03, HM001288.1	99.6
Sole, Moss Landing	Stomach	1014	JQ691539	*Paracoccus* sp. NPO-JL-65, AY745834.1	99.8
		1016	JQ691541	*Psychrobacter okhotskensis* strain MD17, NR_024806.1	99.8
		1007	JQ691542	*Pseudomonas trivialis* strain BIHB 749, DQ885949.1	99.9
	Pyloric caeca	1010	JQ691553	*Microbacterium oxydans*, EU821338.1	99.9
		1015	JQ691554	*Rhodococcus qingshengii* strain KOPRI 25555, HQ824843	100.0
		1013	JQ691540	*Shewanella livingstonensis* strain NF1-17, HM142581.1	100.0
		1008	JQ691535	Uncultured bacterium clone 1103200822058, EU844969.1	99.9
				*Psychrobacter* sp. OW3-RT-1, EF523607.1	99.9
	Midintestine	–	–	–	–
Lantern Fish,Monterey Bay	Entire Intestine	1012	JQ691538	*Bacillus* sp. G1DM-77, EU037267.1	100.0
		1031	JQ691563	*Micromonospora coerulea* DSM 43143, NR_026277	99.6
		1032	JQ691551	*Micromonospora coerulea* DSM 43143, NR_026277	99.6
Lantern Fish,Monterey Bay	Entire Intestine	1009	JQ691537	*Paenibacillus glucanolyticus* strain DSM 5162, NR_040883.1	99.4
		1011	JQ691562	*Paenibacillus glucanolyticus* strain DSM 5162, NR_040883.1	99.5
Red Rock Fish,Seattle	Pyloric caeca	1018	JQ691545	*Rhodococcus* sp. ZS2-21, FJ195998	99.8
		1026	JQ691548	*Dietzia* sp. I_GA_W_11_7, FJ267547.1	99.2
		1033	JQ691552	Uncultured Micromonosporaceae bacterium clone, EU440645	98.3
				*Mycobacterium* sp. 05-Be-043, GU574173.1	98.2
	Pyloric caeca	1021	JQ691556	Actinobacterium CH21i, FJ164059	100.0
	Anterior Intestine	–	–	–	–
	Midintestine	1022	JQ691546	*Rhodococcus* sp. ZS2-21, FJ195998	99.8
	Posterior Intestine	1023	JQ691557	*Micromonospora echinospora* strain 4RS-3a, EU379278	100.0
		1029	JQ691536	*Pseudomonas fragi* strain KOPRI 25817, HQ824990	100.0
Norwegian Mackrel,Seattle	Stomach	1030	JQ691549	*Rhodococcus* sp. ZS2-21, FJ195998	99.9
	Midintestine	–	–	–	–
	Posterior Intestine	–	–	–	–
USA Smelt, Seattle	Entire Intestine	1019	JQ691558	*Micromonospora* sp. R1, EU714258	99.9
		1028	JQ691547	*Arthrobacter* sp. KOPRI 25748, HQ824952.1	99.9
	Roe	1024	JQ691543	*Rhodococcus* sp. ZS2-21, FJ195998	99.8
		1025	JQ691550	*Rhodococcus* sp. Eu-32, DQ386111.2	98.7

Analysis of this phylogenetic tree shows the presence of three main phyla (Actinobacteria, Proteobacteria, and Firmicutes) with three suborders (Corynebacterineae, Micromonosporineae, and Micrococcineae) represented in the Actinobacteria phylum. Interestingly, no isolates from the family Streptomycetaceae were isolated, despite the fact that isolates of the genus *Streptomyces* are regularly obtained from marine sediment samples in our laboratory using the same isolation media. This result is also in contrast to a study of the gut contents of marine ornamental fish, where 87 *Streptomyces* were isolated from homogenized gut contents. [31] Other studies of obligate symbionts from invertebrates and plants have shown that *Streptomyces* are well represented and readily culturable using standard methods. [12], [15] Even in our own laboratory, Actinobacteria enrichment isolations typically yield many different morphotypes from the family Streptomycetaceae. It should also be noted that, under the isolation conditions employed, which include the addition of high concentrations of antifungal and Gram-negative antibiotic agents, each fish yields only a few Actinomycetales strains (Table 1). However, the phylogenetic analysis performed in this study demonstrates that it is possible to enrich for novel Actinobacteria from the fish microbiome.

In selecting BLAST matches for inclusion in the taxonomic analysis we restricted our search to published strains. In some instances, the closest relative to the isolates from the fish microbiome are uncultured bacterial clones (e.g. FI-1033 and FI-1008), with the closest cultured strains having percent sequence identities as low as 98.2%. Examination of the identity of these closest published relatives reveals that several have previously been identified as psychrophilic bacteria, such as the Gammaproteobacteria *Psychrobacter* sp. In fact, many of the closest relatives (12 strains, denoted by blue diamonds in Figure 1) have been isolated from cold environments such as Antarctica, suggesting that fish from temperate marine environments such as the Monterey Bay and Puget Sound, could represent a source of discovery for novel psychrophilic bacterial strains. This is also in contrast to isolates from our sediment-derived microbial isolation program, which rarely results in the isolation of psychrophilic strains, despite being subjected to the same sample storage and isolation methods.

**Figure 2:**
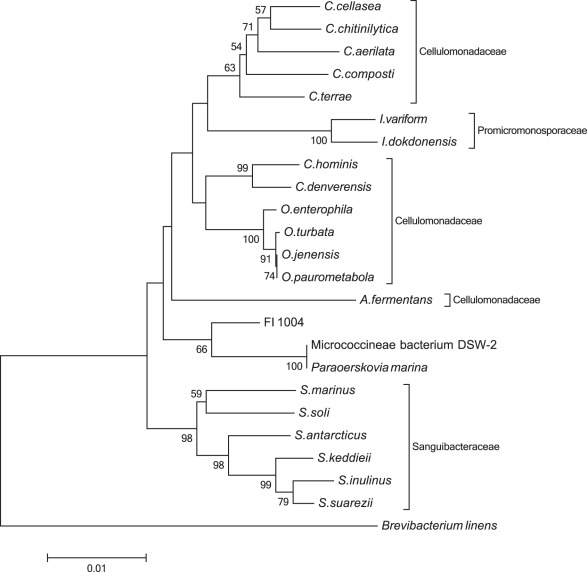
Phylogenetic Relationships of Taxa Related to FI-1004. The evolutionary history was inferred using the Neighbor-Joining method. [50] The bootstrap consensus tree inferred from 1000 replicates is taken to represent the evolutionary history of the taxa analyzed. [49] Branches corresponding to partitions reproduced in less than 50% bootstrap replicates are collapsed. The percentage of replicate trees in which the associated taxa clustered together in the bootstrap test (1000 replicates) are shown next to the branches. [49] The tree is drawn to scale, with branch lengths in the same units as those of the evolutionary distances used to infer the phylogenetic tree. The evolutionary distances were computed using the Maximum Composite Likelihood method [51] and are in the units of the number of base substitutions per site. The analysis involved 24 nucleotide sequences. All positions containing gaps and missing data were eliminated. There were a total of 1353 positions in the final dataset. Evolutionary analyses were conducted in MEGA5. [30] The sequence of *Brevibacterium linens* DSM 20425^T^ was used as an outgroup.

Of the isolated strains, one in particular (FI-1004) showed very low sequence identity to published sequence data (97.8%), suggesting a significant divergence from known cultured isolates. In order to examine the position of this new strain within the existing taxonomic landscape, the 16S rDNA (Table S1) was submitted to the Ribosomal Database Project’s classifier browser. [32] Analysis of the taxonomical hierarchy revealed that FI-1004 is related to the genus *Paraoerskovia*. [33] Using the same database, the 20 type strains with relevance to FI-1004 were identified, and their phylogeny examined using the same parameters as for the original tree (Figure 2). This phylogenetic tree indicates that FI-1004 only clusters with Cellulomonadaceae and Sanguibacteraceae families and loosely clades with *Paraoerskovia marina*. The maximum 16S rDNA sequence similarity of FI-1004 to the three closest type strains is low, at 97.8% for *P. marina* and 97.0% for both *Oerskovia paurometabola* and *O. enterophila*. The *Paraoerskovia* genus was recently described as a novel genus, isolated from a Japanese marine sediment sample, [33] whereas the *Oerskovia* genus, which is closely related to *Cellulomonas* genus, is typically isolated from environmental soil samples. [34]

One of the goals within our laboratory is the discovery and development of lead compounds for drug development against protozoan parasites and bacterial targets. To examine the capacity of these isolates to produce bioactive natural products, 21 of these isolates were selected based on morphological characteristics, and large-scale liquid cultures prepared and extracted using our standard protocol. Crude extracts were prefractionated on C_18_ cartridges to give seven prefractions, and screened against a panel of human and fish bacterial pathogens.

In both wild and farmed fish, vibriosis is one of the most common bacterial diseases to affect fish populations. Vibriosis is caused by a number of related *Vibrio* species, all of which are fast growing opportunistic bacteria that can quickly dominate the intestinal microbiota of fish. [35] It is estimated that all marine fish are susceptible to infection by at least one species of *Vibrio*, [36] however, the number of therapeutic options designed specifically for fish pathogens is low, leading to frequent over use of traditional antibiotics in aquaculture. The development of new compounds that specifically target marine *Vibrio* species could provide an alternative approach to aquaculture stock management that would remove or reduce the current dependence on antibiotics used in human health applications, which in turn would have a positive effect on antibiotic management strategies.

The results from growth inhibition assays against this panel of bacterial pathogens are summarized in Table 2. Overall, nine of the 21 extracts showed activity against one or more target strains, with five of the nine active extracts inhibiting the growth of one or more Gram-positive strains, and eight of the nine inhibiting the growth of one or more Gram-negative strains. This is in contrast to traditional natural product libraries, where Gram-positive antibiotics are typically much more common than those that target Gram-negative strains. [37] Of the eight *Vibrio* species in the panel, five were inhibited by one or more extracts, with most active extracts showing activity against two or more *Vibrio* species. In addition, three of the eight *Vibrio* strains were inhibited by two or more extracts, suggesting that some of these compounds may have broad spectrum efficacy for treatment of vibriosis in aquaculture facilities.

**Table 2:** Bioactivities of Fish Microbiome Isolates.

Strain	Phylum	*V. fischeri*(−)	*V. cholerae*(−)	*V. hollisae*(−)	*B. subtilis*(+)	*V. mimicus*(−)	*S. aureus*(+)	*Y. ruckeri*(−)	*E. faecium*(+)	*V. vulnificus*(−)
FI-1001	A									
FI-1002	A									
FI-1003	A									
FI-1004	A				✓	✓	✓		✓	
FI-1005	A		✓							✓
FI-1006	A									
FI-1007	Γ									
FI-1008	Γ					✓				
FI-1009	F							✓	✓	
FI-1010	A	✓				✓			✓	
FI-1011	F									
FI-1012	F									
FI-1013	Γ	✓	✓	✓	✓	✓	✓	✓		
FI-1014	Α									
FI-1015	A									
FI-1016	Γ									
FI-1017	F				✓		✓		✓	
FI-1018	A									
FI-1019	A		✓			✓				
FI-1020	A		✓			✓				
FI-1021	A									

A = Actinobacteria, F = Firmicutes, α = Alphaproteobacteria, γ = Gammaproteobacteria, (−) = Gram-negative, (+) = Gram-positive. Check mark indicates activity in growth inhibition assay.

**Figure 3:**
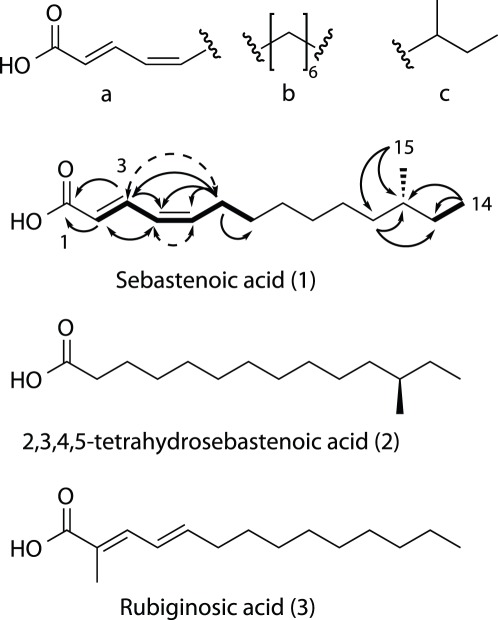
Chemical Structure for Sebastenoic Acid. a, b and c are subunits found using 2D NMR methods. HMBC correlations depicted by solid arrows, COSY correlations depicted by bold lines, NOESY correlations depicted by dashed arrows.

Of the extracts that showed activity in this screen, FI-1004 was of particular interest because of its low sequence identity to other published bacterial sequences (*vide supra*). Semipreparative reverse phase high performance liquid chromatography (RP-HPLC) yielded five fractions for secondary screening. Screening of these sub-fractions revealed a single major component of the mixture that was responsible for the observed bioactivity. Purification of this material using C_18_ RP-HPLC afforded a novel bioactive lipid, sebastenoic acid (**1**), as an optically active yellow solid (Figure 3).

Structure elucidation for this new metabolite was performed as follows. HRESI-TOFMS analysis gave the molecular ion [M+Na]^+^ at 261.1835 which was consistent with the molecular formula C_15_H_26_O_2_Na (calcd. 261.1825). The UV spectrum for sebastenoic acid implied the presence of an extended conjugated system (λ_max_ = 268 nm), which was supported by the presence of four olefinic ^13^C resonances (δ 121.7, 127.1, 140.8, 142.8) and one carboxylic acid carbonyl ^13^C resonance (δ 167.9). COSY and HMBC correlations indicated that these motifs were connected as an α-β-γ-δ unsaturated carboxylic acid moiety (Figure 3), which was supported by the chemical shift values for the olefinic carbon resonances. The H2 – H3 *^3^J_HH_* coupling constant had a large value (15.0 Hz), assigning the C2 olefin as *E*. The H4 – H5 *^3^J_HH_* coupling constant had a smaller value (10.8 Hz), suggesting that the C4 olefin possesses the *Z* geometry. This was confirmed by NOESY correlations between H4 and H5, and between H3 and H6, which can only be accounted for if the C4 olefin possesses the cis conformation.

To assign the remainder of the molecule, we turned to the ^1^H NMR spectrum (Figures S1, S2, S3, S4, S5, and S6), which revealed the presence of two methyl groups (δ 11.6, d, *J* = 6.8 Hz; δ 19.5, t, *J* = 7.2 Hz) and a large methylene envelope (δ 1.27–1.34) suggesting the presence of a methyl branched alkyl chain. Key HMBC correlations from H12 to C11, C14 and C15 allowed the placement of methyl group C15 at the antepenultimate carbon of the alkyl chain, forming a sec-butyl motif, and assigning sebastenoic acid an anteiso fatty acid (Figure 3). The remaining unassigned atoms possessed the formula C_5_H_10_, which could only be assigned as a linear methylene chain based on the ^13^C multiplicity obtained from the DEPT-135 spectrum. Assembly of these three subunits gave the planar structure of sebastenoic acid as (2*E*,4*Z*)-12-methyltetradeca-2,4-dienoic acid (Table 3).

**Table 3:** NMR Chemical Shift Table for Sebastenoic Acid.

Position	δ_c_, Multiplicity	δ_H_	^1^H Multiplicity(*J* in Hz)
1	167.9, C	–	–
2	121.7, CH	5.86	d, 15.0
3	140.8, CH	7.57	dd,15.0, 11.4
4	127.1, CH	6.18	dd, 11.4, 10.8
5	142.8, CH	5.91	dt,10.8, 7.8
6	28.7, CH_2_	2.30	ddd, 8.4, 7.8, 1.2
7	29.9, CH_2_	1.43	m
8	27.7,^a^ CH_2_	1.34–1.27	m
9	29.8,^a^ CH_2_	1.34–1.27	m
10	30.4,^a^ CH_2_	1.34–1.27	m
11	30.1, CH_2_	1.14, 1.30	m
12	35.1, CH	1.31	m
13	37.2, CH_2_	1.10, 1.32	m
14	19.5, CH_3_	0.85	t, 7.2
15	11.6, CH_3_	0.86	d, 6.8

a Assignments interchangeable.

Determination of the absolute configuration of the chiral center at C-12 was based on comparison with the reported optical rotation for the hydrogenated *a*15∶0 fatty acid. [38], [39] Natural sebastenoic acid was reduced by hydrogenation over 10% palladium on carbon to give 2,3,4,5-tetrahydrosebastenoic acid (**2**). Comparison of the optical rotation for **2** ([α]_D_^24^ = −7.21) with the reported literature value for (*R*)-12-methyltetradecanoic acid ([α]_D_^22^ = −5.84) defined the absolute configuration at C12 as *R*.

Branched chain fatty acids are ubiquitous in the bacterial world and are common substrates for phospholipid synthesis, these lipids typically being incorporated into bacterial cell membranes. [39] In addition, saturated anteiso fatty acids are frequently encountered in nature, however unsaturated anteiso fatty acids containing 15 carbon atoms are comparatively rare, with only one previous example having being reported. [40] (2*E*,4*E*)-2-Methyl-tetradeca-2,4-dienoic acid (**3**) (rubiginosic acid), another α-β-γ-δ-unsaturated fatty acid, was isolated from the terrestrial fungus *Hypoxylon rubiginosu*, however no antibacterial activity was reported.

Biological testing of **1** against relevant test organisms gave MIC values as summarized in Table 4. Overall, **1** showed reasonable activity against the three Gram-positive test strains, with low activity against the Gram-negative test strain. The ability of strain FI-1004 to produce an antibiotic with activity against common pathogens could indicate its capacity as a probiotic constituent of the microbiome of the fish host. Colonization of the fish intestinal tract occurs just hours after hatching, and it has been demonstrated that maintaining a healthy microbiota is a key factor in maintaining overall health of host organisms. [41] Correct establishment of the microbiota in fish is critical to survival, as they are in direct contact with pathogenic and opportunistic bacteria in the marine environment. There is evidence that probiotic bacteria afford greater benefit to the health of farmed fish than commercially available products. [41] Understanding the innate microbial composition of commercial stock species is key in determining appropriate probiotics for protection against harmful bacteria. Along these lines, the development of new antibiotics or probiotics will be of significant value to the aquaculture industry, which is the fastest growing food production sector according to the Food and Agriculture Organization. [42].

**Table 4:** Sebastenoic Acid (1) MICs Against Bacterial Panel.

	*B. subtilis*	*S. aureus*	*V. mimicus*	*E. faecium*
MIC (µg/mL)	11.2	23.8	110.6	10.0

In conclusion, we have shown that the fish microbiome represents a unique source of microbial diversity, and that these strains have the capacity to produce novel bioactive secondary metabolites. Isolated strains were shown to possess activity against both Gram-positive and Gram-negative bacterial pathogens. Examination of the chemical constituents from one of these extracts lead to the isolation of the novel anteiso fatty acid sebastenoic acid (**1**), which possesses a unique unsaturation pattern for this compound class.

The ability of the isolated cultures to produce compounds which demonstrate activity against fish pathogens, such as *Vibrio* spp., suggest that bacteria isolated from the fish microbiome could be used as probiotic agents, and that they could be play a role in innate microbial biocontrol in wild populations. Overall, this research represents the first investigation into the discovery of natural products from the fish microbiome.

## Materials and Methods

### Fish Collection and Dissection

Whole dead intact fish were purchased from commercial vendors in Moss Landing, California and Seattle, Washington, and stored at −20°C until workup. All instruments, surfaces, and the exterior of each fish were treated with 70% EtOH and instruments were flame sterilized prior to dissection. The dissection was performed in the presence of a flame, and instruments were flame sterilized after each cut. An incision was made on the ventral side of each fish at the anus, and extended anteriorly to the isthmus. A second cut was made dorsally through the operculum. The stomach, pyloric caeca and intestines were separated from the body cavity and sterilized with 70% EtOH. The contents of the digestive tract were transferred to a sterile 10 mL Falcon tube at discrete portions of the gut: the posterior portion of the intestine, the mid-intestine, the stomach, and the pyloric caeca, as appropriate for each specimen.

### Cultivation of Bacteria

Fish intestine contents were transferred to sterile Falcon tubes, 1 mL of sterile Milli-Q water was added, and the samples vortexed for 1 minute. Four different solid agar media were used for microbial isolation: actinomycete isolation agar (Difco), SNS, [43] and modified NTS and HVS. [44] All isolation plates were prepared with sterile sea water and supplemented with 50 mg/L of both cyclohexamide and nalidixic acid. Intestinal contents were plated using three different methods: a) the mixture was serially stamped onto solid agar with a sterile swab, b) the mixture was diluted with 1 mL of sterile Milli-Q water and 100 µL of the resulting mixture spread onto the plate surface, c) the mixture was diluted with 10 mL of sterile Milli-Q water and 100 µL of the resulting mixture spread onto the plate surface. Cultures were incubated at room temperature and bacterial colonies displaying desired morphologies subcultured on Difco Marine Broth solid agar plates until pure. Typical incubation times for the appearance of colonies from isolation plates ranged from 30–90 days.

### DNA Isolation, PCR Amplification and Sequencing

Genomic DNA was extracted using Microlysis buffer (Gel Company) by picking a single colony of the cultured strains according to manufacturers instructions. For PCR amplification of 16S rRNA gene, the primer pair 8F (5′-AGAGTTTGATCCTGGCTCAG-3′) and 1492R (5′-GGTTACCTTGTTACGACTT-3′) was used. [45] Each reaction contained a total volume of 25 µL (2.0 µL genomic DNA, 1.0 µL of a 10 µM solution for each primer, 8.5 µL sterile water and 12.5 µL of MegaMix-Gold (Gel Company)). PCR was performed on a Eppendorf Mastercycler Personal thermocycler under the following conditions: initial denaturation 95°C for 5 min, 35 cycles of denaturation at 95°C for 1 min, annealing at 50°C for 1 min, extension at 72°C for 1 min and 30 sec, and a final extension at 72°C for 10 min. After confirmation by gel electrophoresis (1% agarose gel in 1x TAE buffer), the PCR products were purified with QIAQuick PCR Purification Kit (Qiagen) and sent directly to Sequetech Corporation for sequencing using the same PCR primers described above plus an additional middle primer 341F (5′-CCTACGGGAGGCAGCAG-3′). [46] DNA sequences were deposited to GenBank with accession numbers JQ691535-JQ691563.

### Extraction of Cultivated Isolates

Purified bacterial colonies were grown in 1 L of modified SYP broth [47] (1L MilliQ water, 32.1 g Instant Ocean™, 10 g starch, 4 g peptone, 2 g yeast) with 20 g of Amberlite XAD-16 resin for 10 days at 27°C. Culture broth and resin slurries were filtered through glass microfiber filters, washed with water (3×200 mL) and the cells, resin, and filter paper extracted with 1∶1 methanol/dichloromethane (250 mL). Organic fractions were dried *in vacuo* and subjected to solid phase extraction (SPE) using a Supelco-Discovery C_18_ cartridges (5 g) eluting with a step gradient of 40 mL of MeOH/H_2_O solvent mixtures (10% MeOH, 20% MeOH, 40% MeOH, 60% MeOH, 80% MeOH, 100% MeOH) and finally with EtOAc to afford seven fractions. The resulting fractions were dried *in vacuo*, resolubilized in 500 µL of dimethyl sulfoxide (DMSO), and transferred to deep well 96-well plates for screening.

### Growth Inhibition Assay

Overnight saturated cell cultures of pathogenic strains (Text S1) were diluted 1∶1000 with fresh media and 30 µL of culture dispensed into each well of sterile clear 384-well plates. 300 nL of DMSO prefraction stock solutions were pinned into screening plates using a Perkin Elmer Janus MDT robot. After inoculation, screening plates were stacked in a plate reader/shaker (Perkin Elmer EnVision) and OD_600_ readings taken once per hour for 24 hrs. Computer generated growth curves for serially diluted pure compounds were used to determine MIC values by correlating the OD_600_ reading at the pre-exponential phase of the bacteria to the concentrations in individual wells.

### General Experimental Procedures

Unless otherwise stated, reactions were performed under an argon atmosphere using freshly dried solvents. Methylene chloride (DCM) was dried by passing through an activated alumina column. Solvents used for HPLC chromatography were HPLC grade and were used without further purification. Optical rotations were measured on a Jasco P-2000 polarimeter using a 10 mm path length cell at 589 nm. NMR spectra were acquired on a Varian Inova 600 MHz spectrometer equipped with a 5 mm HCN triple resonance cryoprobe, and referenced to residual solvent proton and carbon signals (δH 7.26, δC 77.16 for CDCl_3_ and δH 1.94, δC 1.39 for CD_3_CN). High-resolution mass spectra were acquired with an ABI Mariner ESI-TOF-MS.

### Extraction and Isolation

Seed culture of FI-1004 was grown in 1 L of modified SYP broth with 20 g of Amberlite XAD-16 resin for 10 days at 27°C and the organic extract generated as described above. The active 80% MeOH fraction was subjected to C_18_ RP-HPLC Phenomenex Jupiter C18 (4.6×250 mm, 5 µm), 82% MeOH/18% H_2_O (acidified with 0.002% formic acid), 1 mL/min, 254 nm, t_R_ = 16.6 to give sebastenoic acid as an optically active yellow solid (Figure S7).

### Hydrogenation of Sebastenoic Acid

To a stirred solution of **1** (0.55 mg, 2.31 µmol) in dry DCM (1.5 mL) was added 10% palladium on carbon (5 mg) and the resulting suspension stirred under an atmosphere of H_2_ (balloon) at room temperature for 16 h. The sample was concentrated to dryness under a stream of N_2_, dissolved in MeOH (1 mL), and filtered through a 13 mm 0.2 µm nylon filter. The resulting filtrate was concentrated to dryness under a stream of N_2_ to give 2,3,4,5-tetrahydrosebastenoic acid (**2**) as a white solid (0.45 mg, 81% yield).

### Compound Characterization

**Sebastenoic acid ((2** ***E*** **,4** ***Z*** **)-12-methyltetradeca-2,4-dienoic acid) (1)**: yellow solid; [α]_D_^24^ −8.91 (*c* 0.145, CHCl_3_); UV (CHCl_3_) λ_max_ (log ε) 268 nm (4.31); for ^1^H and ^13^C NMR data see Table 3; HRESITOFMS m/z [M+Na]^+^ 261.1835 (calcd for C_15_H_26_O_2_Na, 261.1825).

**2,3,4,5-Tetrahydrosebastenoic acid (2)**: yellow solid; [α]_D_^24^ −7.21 (*c* 0.41, CHCl_3_); ^1^H NMR (CDCl_3_, 600 MHz) δ 2.36 (t, *J* = 7.8 Hz, 2H), 1.64 (p, *J* = 7.8 Hz, 2H), 1.34−1.24 (m, 17H), 1.15−1.06 (m, 2H), 0.85 (t, *J* = 7.2 Hz, 3H), 0.84 (d, *J* = 6.8 Hz, 3H); HRESIFTMS *m/z* [M – H]^−^ 241.2174 (calcd for C_15_H_29_O_2_, 241.2173).

## Supporting Information

Text S1 **Panel of Bacterial Strains.**
(DOCX)

Figure S1 **Sebastenoic Acid ^1^H, d3-MeCN, 600 MHz.**
(TIF)

Figure S2 **Sebastenoic Acid ^13^C, d3-MeCN, 600 MHz.**
(TIF)

Figure S3 **Sebastenoic Acid gCOSY, d3-MeCN, 600 MHz.**
(TIF)

Figure S4 **Sebastenoic Acid HMQC, d3-MeCN, 600 MHz.**
(TIF)

Figure S5 **Sebastenoic Acid gHMBC, d3-MeCN, 600 MHz.**
(TIF)

Figure S6 **Sebastenoic Acid gNOESY, d3-MeCN, 600 MHz.**
(TIF)

Figure S7 **Isolation Scheme for Sebastenoic Acid and Bacterial Colony Photograph.**
(TIF)

Table S1 **Strains and Accession number for** **Figure 2** **.**
(XLSX)
