# Supplementary material for: Examining the Fish Microbiome: Vertebrate-Derived Bacteria as an Environmental Niche for the Discovery of Unique Marine Natural Products
Source: PLoS One. 2012 May 4;7(5):e35398. doi: 10.1371/journal.pone.0035398 (PMC3344833; doi:10.1371/journal.pone.0035398)
Supplement: Text S1 — Panel of Bacterial Strains. (DOCX) [file pone.0035398.s001.docx]

**Text S1. Panel of Bacterial Strains**

Screening panel consists of 9 bacterial strains including some biosafety level 2 pathogens that were selected to offer broad clinical relevance. The Gram-positive strains include *Bacillus subtilis* 168*,*[1] *Staphylococcus aureus* (ATCC 29213)*,* and *Enterococcus faecium* (ATCC 6569) while the gram-negative strains include *Yersina ruckeri* (ATCC 29473)*, Vibrio cholerae* O1 (biotype El Tor A1552), [2], *Vibrio vulnificus*,[3] *Vibrio mimicus*,[3] *Vibrio fischeri* MJ1,[4] and *Vibrio hollisae*.[3]

*Staphylococcus aureus, Y. ruckeri,* and *E. faecium* cultures were grown in 10 mL of tryptic soy broth (17 g tryptone, 3 g soytone, 2.5 g dextrose, 5 g NaCl and 2.5 g dipotassium phosphate in 1 L distilled water; pH 7.5). The *B. subtilis,* and all Vibrio cultures were grown in Luria Broth (10 g tryptone, 5 g yeast extract and 10 g NaCl in 1 L distilled water; pH 7.5). Both media were autoclaved at 121 °C for 30 min. Inoculated cultures were grown overnight under shaking conditions (200 rpm; 30 °C). Overnight saturated cell cultures of pathogenic strains were diluted 1:1000 with fresh media and 30 μL of culture dispensed into each well of sterile clear bottom 384-well plates.

1. Carballido-Lopez R, Formstone A, Li Y, Ehrlich SD, Noirot P, et al. (2006) Actin homolog MreBH governs cell morphogenesis by localization of the cell wall hydrolase LytE. Dev Cell 11: 399-409.

2. Yildiz FH, Schoolnik GK (1999) Vibrio cholerae O1 El Tor: Identification of a gene cluster required for the rugose colony type, exopolysaccharide production, chlorine resistance, and biofilm formation. Proc Natl Acad Sci USA 96: 4028-4033.

3. California Department of Public Health, Microbial Disease Laboratory.

4. Ruby EG, Nealson KH (1976) Symbiotic Association of Photobacterium-Fischeri with Marine Luminous Fish Monocentris-Japonica - Model of Symbiosis Based on Bacterial Studies. Biol Bull 151: 574-586.
