# Supplementary figures and images for: Examining the Fish Microbiome: Vertebrate-Derived Bacteria as an Environmental Niche for the Discovery of Unique Marine Natural Products
Source: PLoS One. 2012 May 4;7(5):e35398. doi: 10.1371/journal.pone.0035398 (PMC3344833; doi:10.1371/journal.pone.0035398)

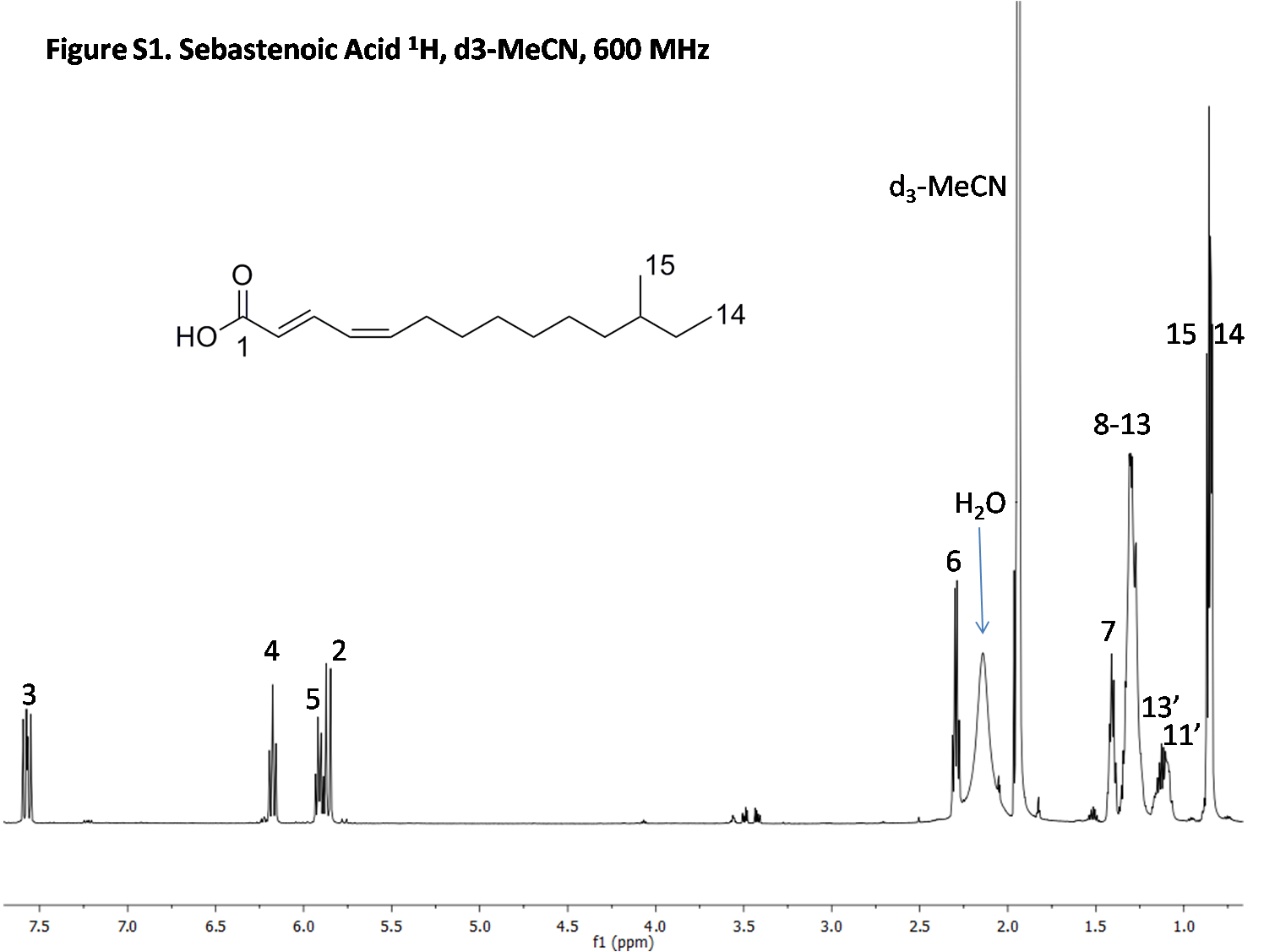

Supplement: Figure S1 — Sebastenoic Acid 1H, d3-MeCN, 600 MHz. (TIF) [file pone.0035398.s002.tif]

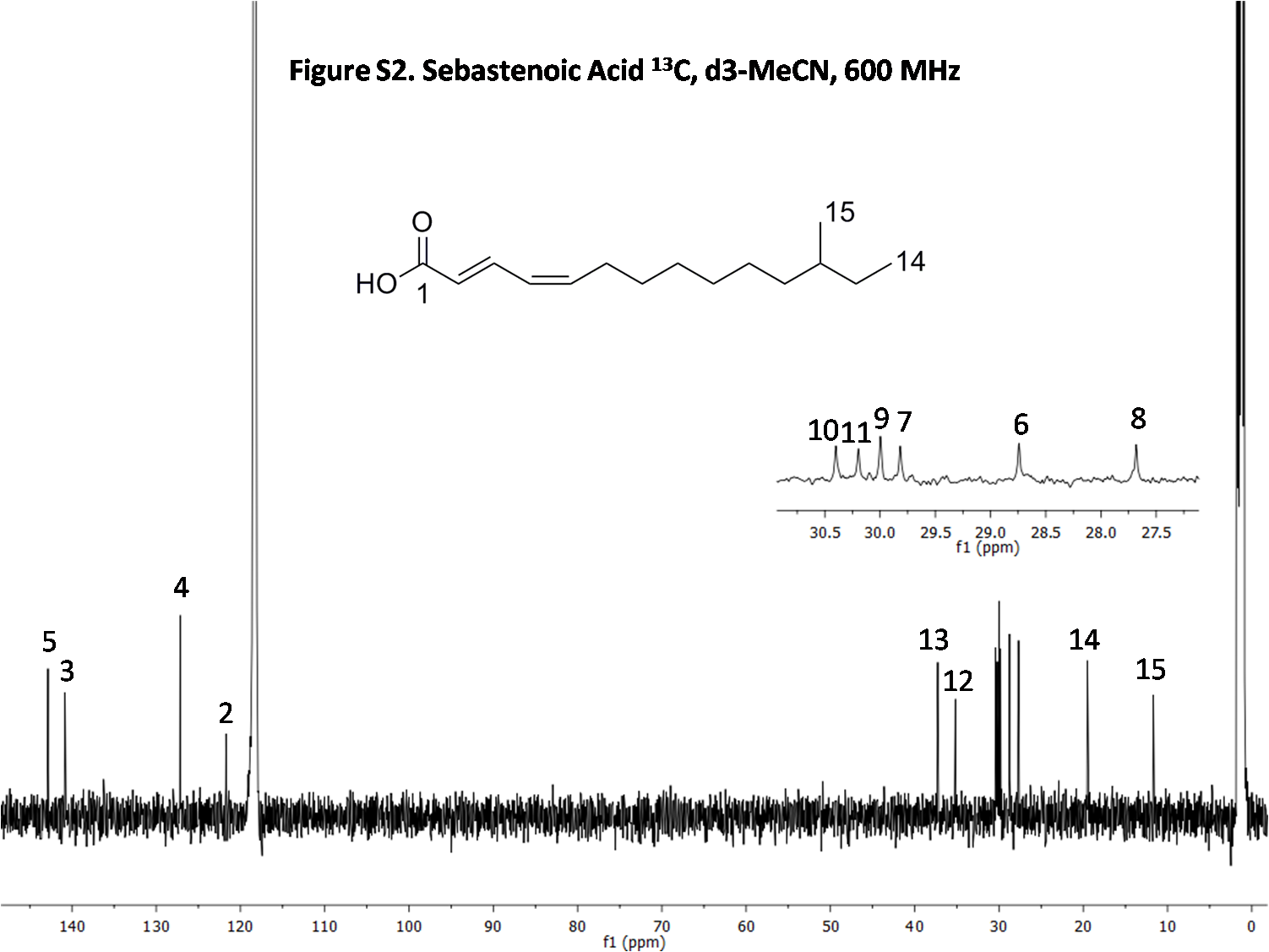

Supplement: Figure S2 — Sebastenoic Acid 13C, d3-MeCN, 600 MHz. (TIF) [file pone.0035398.s003.tif]

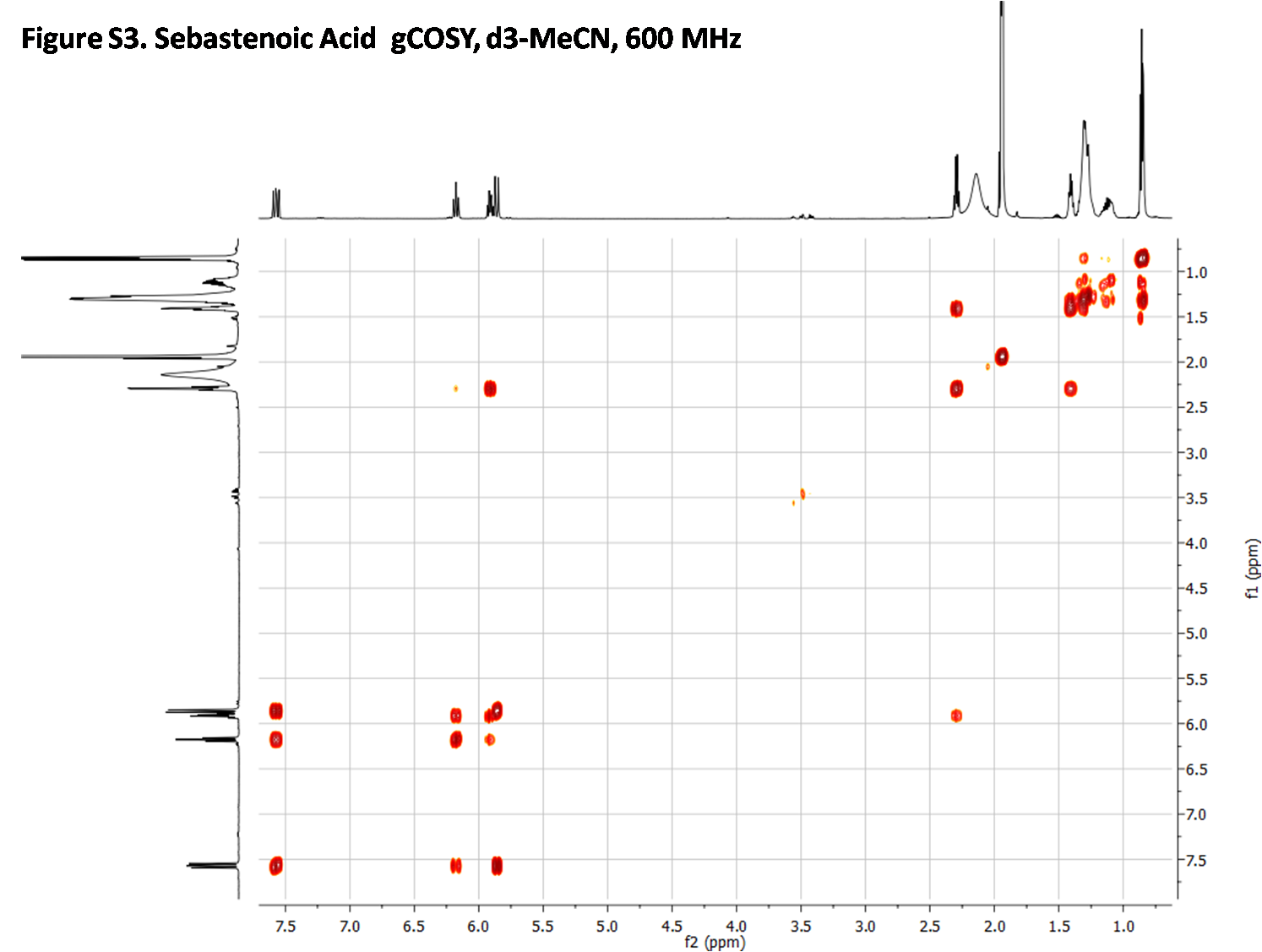

Supplement: Figure S3 — Sebastenoic Acid gCOSY, d3-MeCN, 600 MHz. (TIF) [file pone.0035398.s004.tif]

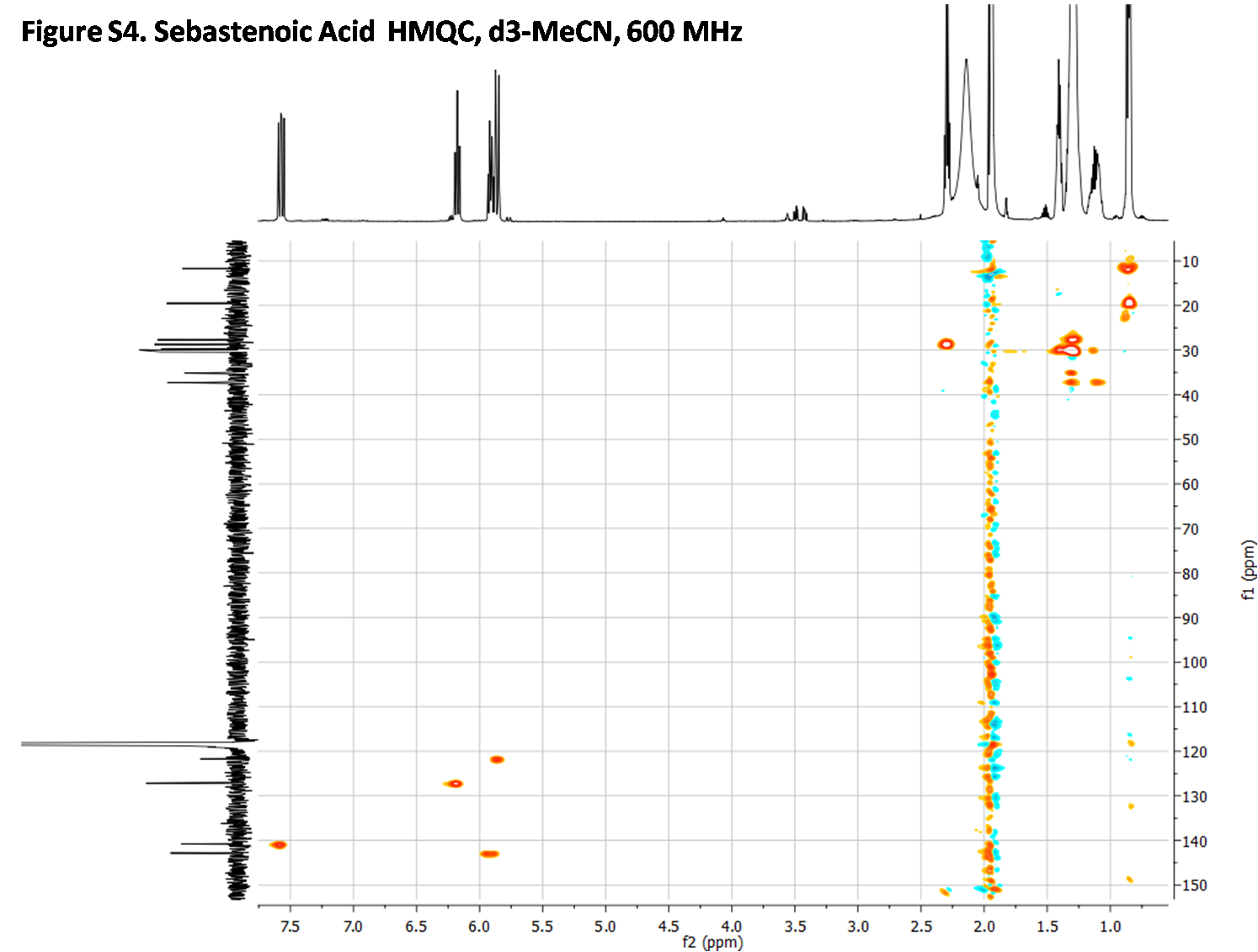

Supplement: Figure S4 — Sebastenoic Acid HMQC, d3-MeCN, 600 MHz. (TIF) [file pone.0035398.s005.tif]

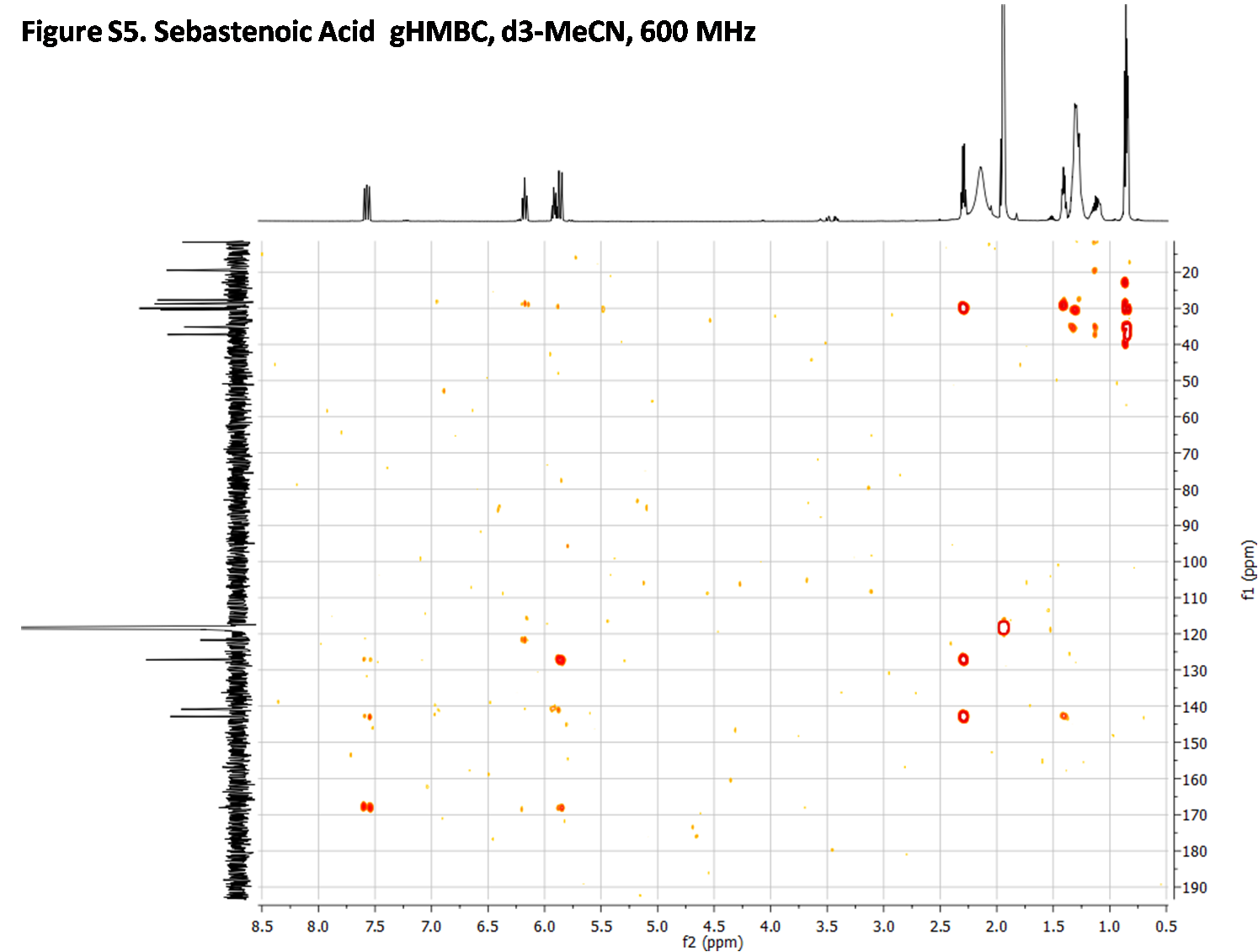

Supplement: Figure S5 — Sebastenoic Acid gHMBC, d3-MeCN, 600 MHz. (TIF) [file pone.0035398.s006.tif]

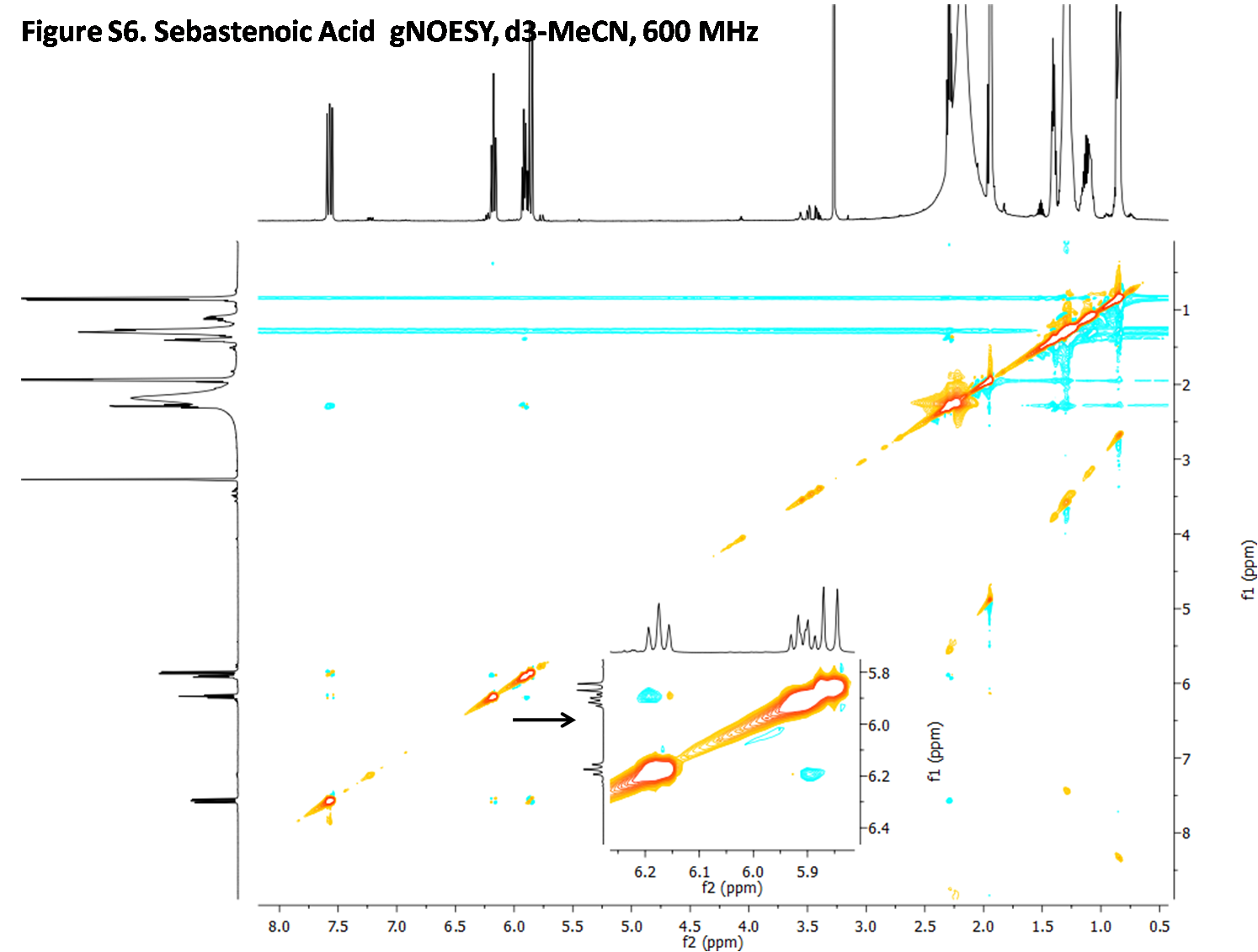

Supplement: Figure S6 — Sebastenoic Acid gNOESY, d3-MeCN, 600 MHz. (TIF) [file pone.0035398.s007.tif]

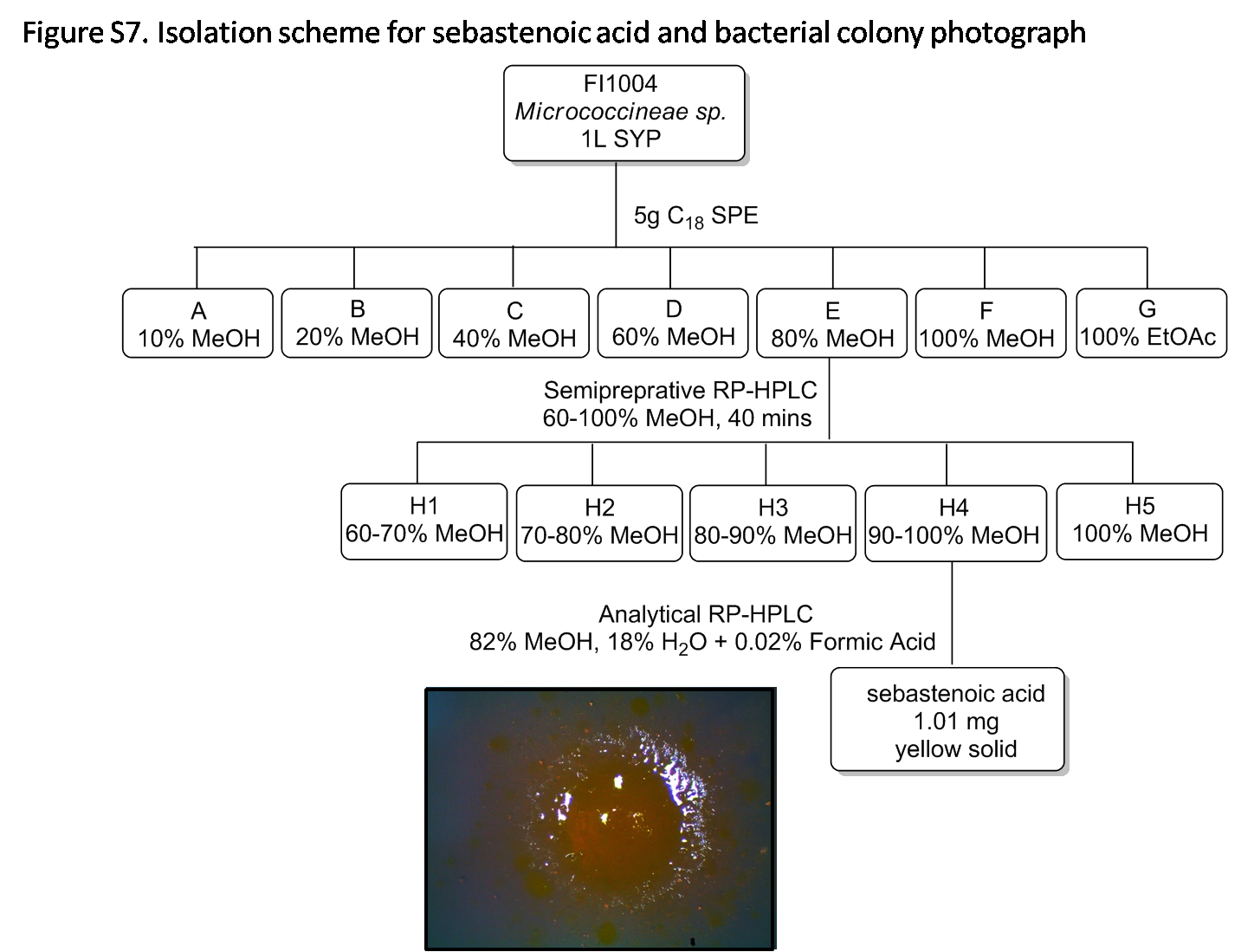

Supplement: Figure S7 — Isolation Scheme for Sebastenoic Acid and Bacterial Colony Photograph. (TIF) [file pone.0035398.s008.tif]
